# Caspase-1 Promotes Epstein-Barr Virus Replication by Targeting the Large Tegument Protein Deneddylase to the Nucleus of Productively Infected Cells

**DOI:** 10.1371/journal.ppat.1003664

**Published:** 2013-10-10

**Authors:** Stefano Gastaldello, Xinsong Chen, Simone Callegari, Maria G. Masucci

**Affiliations:** Department of Cell and Molecular Biology, Karolinska Institutet, Stockholm, Sweden; University of Glasgow, United Kingdom

## Abstract

The large tegument proteins of herpesviruses contain N-terminal cysteine proteases with potent ubiquitin and NEDD8-specific deconjugase activities, but the function of the enzymes during virus replication remains largely unknown. Using as model BPLF1, the homologue encoded by Epstein-Barr virus (EBV), we found that induction of the productive virus cycle does not affect the total level of ubiquitin-conjugation but is accompanied by a BPLF1-dependent decrease of NEDD8-adducts and accumulation of free NEDD8. Expression of BPLF1 promotes cullin degradation and the stabilization of cullin-RING ligases (CRLs) substrates in the nucleus, while cytoplasmic CRLs and their substrates are not affected. The inactivation of nuclear CRLs is reversed by the N-terminus of CAND1, which inhibits the binding of BPLF1 to cullins and prevents efficient viral DNA replication. Targeting of the deneddylase activity to the nucleus is dependent on processing of the catalytic N-terminus by caspase-1. Inhibition of caspase-1 severely impairs viral DNA synthesis and the release of infectious virus, pointing a previously unrecognized role of the cellular response to danger signals triggered by EBV reactivation in promoting virus replication.

## Author Summary

Viruses rely on the host cell for replication and have evolved sophisticated strategies to manipulate and harness the cellular metabolic pathways and defense responses. A better knowledge of these viral strategies will provide new targets for antiviral therapies. The N-terminus of the large tegument proteins of herpesviruses encodes an ubiquitin and NEDD8-specific deconjugase, but the function of the enzyme during virus replication is largely unknown. Here we report that, endogenously expressed BPLF1, the homolog encoded by Epstein-Barr virus (EBV), promotes a dramatic decrease of NEDD8-conjugates and the accumulation of free NEDD8 in cells entering the productive virus cycle. BPLF1 exerts its deneddylase activity in the nucleus, which promotes the accumulation of cullin-RING ligase (CRL) substrates that are required for efficient virus replication. Targeting of the viral enzyme to the nucleus is dependent on processing of the catalytic N-terminus by caspase-1. Inhibition of caspase-1 severely impairs viral DNA synthesis and the release of infectious virus, pointing to an unexpected role of the cellular response to danger signals triggered by EBV reactivation in promoting virus replication.

## Introduction

Post-translational modification of proteins by covalent linkage of ubiquitin (Ub) or ubiquitin-like proteins (UbLs), such as SUMO, NEDD8, ISG15, regulates diverse cellular processes, including cell cycle progression, DNA repair, transcription, signal transduction and immune responses [1], [2]. Cytosolic and nuclear proteins tagged with multiple Lys48-linked Ub moieties are targeted to the proteasome for degradation, whereas the attachment of single or multiple Ub or UbLs regulates a variety of non-proteolytic events, including protein-protein interactions and intracellular traffic [3]. Conjugation of the modifiers is accomplished by an enzymatic cascade composed of activating enzymes (E1), conjugating enzymes (E2) and substrate-specific ligases (E3), and is reversed by substrate-specific cysteine or metallo-protease that control the turnover of the modification and play thereby a key role in determining the functional outcome. Although each modifier is involved in unique cellular functions, important cross-talk has been highlighted by the demonstration that NEDD8 and SUMO regulate the activity of certain ubiquitin ligases. Thus, the best characterized substrates of NEDD8 conjugation are cullins that function as scaffolds for the assembly of cullin-RING ubiquitin ligases (CRLs) [4], [5], while SUMOylation is required for substrate recognition and subsequent ubiquitination by a family of SUMO-targeted ubiquitin ligases (STUbLs) [6]. Furthermore, several deconjugases, including USP21 [7], Ataxin-3 [8] PfUCH54 [9], UCH-L1 and UCH-L3 [10], exhibit dual specificity for Ub and NEDD8 conjugates, while SENP8 is both a NEDD8 and SUMO deconjugase [11]. The significance of these multiple specificities in the context of biological processes remains, however, undefined.

Viruses rely on the host cell machinery for replication. Given the key role of Ub and UbL modifications in the regulation of cellular and immunological functions, modulation of these signaling pathways is essential for viral DNA synthesis and for the survival of the virus during acute, chronic and latent infections [12]. Two viral interference strategies have been documented in the infected cells. Viral proteins were shown to regulate the expression or capture the activity of cellular components of the Ub and UbL signaling networks and to redirect their function towards preferred cellular or viral substrates [13]. In addition, there are numerous examples of virus-encoded homologs of cellular ligases and deconjugases [14]. These viral enzymes are often multifunctional proteins that share little homology with their cellular counterparts and are therefore attractive targets for selective inhibition.

Adenovirus [15], severe acute respiratory syndrome coronavirus [16], and all members of the herpesvirus family [17], [18], encode their own deconjugase. The homologs encoded in the N-terminus of the large tegument proteins of herpesviruses show very little sequence similarity but the residues implicated in the formation of the catalytic site are strictly conserved. All the tested homologs have potent ubiquitin-specific protease activity and their overexpression in transfected cells is associated with a dramatic decrease of polyubiquitinated substrates [17], [18]. Expression of the active enzymes was confirmed during infection by human CMV (HCMV) [19], [20], murine gamma-herpesvirus 68 (MHV-68) [12], Marek's disease virus (MDV) [21], and pseudorabies virus (PrV) [22]. Although not essential for viral DNA synthesis, disruption of the catalytic function correlated with severe impairment of viral replication in *in vivo* models of infection [12], [21], [22]. The Epstein-Barr virus (EBV) encoded homolog, BPLF1, has very potent ubiquitin deconjugase activity in various experimental models. Anchoring the enzymatic domain to the ER membrane [23] or injection of the purified protein in semi-intact cells [24] promoted the dislocation of ubiquitinated ERAD substrates, resulting in their stabilization in the cytosol. Overexpression of the N-terminus was associated with deubiquitination of the viral ribonucleotide reductase (RR) [25] and the cellular DNA polymerase processivity factor PCNA [26], resulting in downregulation of the viral RR activity and attenuation of Polη at DNA damage sites. Furthermore, expression of the catalytically active BPLF1 was shown to correlate with deubiquitination of TRAF6 and inhibition of NF-κB signaling during productive EBV infection [27]. We have previously reported that, in addition to their deubiquitinating activity, BPLF1 and the homologs encoded by HSV1, KSHV and MHV-68 exhibit strong activity against NEDD8 conjugates [28]. BPLF1 hydrolyzes NEDD8 conjugates *in vitro* and stabilizes several CRL substrates in transfected cells, including the cellular DNA-replication licensing factor CDT1. Expression of BPLF1 alone or in the context of the productive virus cycle induced the accumulation of CDT1 and arrest of the cells in S-phase, while re-expression of CDT1 was sufficient to revert the inhibition of virus replication induced by BPLF1 knockdown. This phenotype is dependent on direct binding of BPLF1 to CRLs via interaction of the conserved helix-2 of BPLF1 with the C-terminal domain (CTD) of cullins, at a site that is also engaged by the CRL regulator CAND1 [29].

While the double specificity of this family of viral enzymes is experimentally documented, the importance of the ubiquitin and NEDD8-specific deconjugase activities in infected cells is not easily understood since deubiquitination or inactivation of the specific ubiquitin ligase may have similar effects on individual substrates. Thus, it remains unclear whether both the ubiquitin- and NEDD8-specific deconjugase activities operate during virus replication and, if so, how the different functions are regulated or compartmentalized in the infected cells. Here we report that induction of the productive virus cycle has no appreciable effects on the global levels of protein ubiquitination but is accompanied by a BPLF1-dependent decrease of cullin neddylation and stabilization of nuclear CRL substrates. Targeting of catalytic N-terminus of BPLF1 to the nucleus is dependent on cleavage by caspase-1, and inhibition of the caspase halts virus replication and the release of infectious virus.

## Results

### Protein neddylation is selectively affected during EBV replication

**Figure 1:**
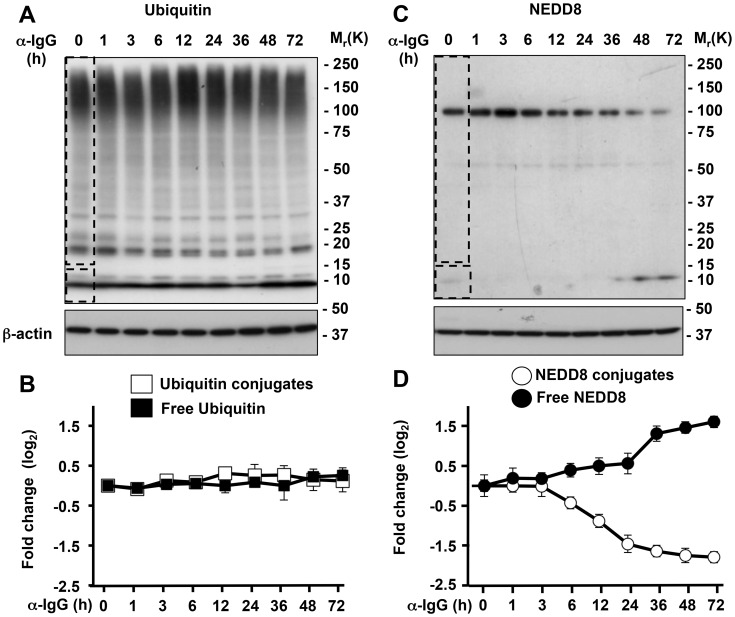
Neddylation is selectively impaired during EBV replication. Western blots of cell lysates from induced Akata-Bx1 were probed as indicated. **A**. Western blot illustrating the unchanged levels of ubiquitin conjugates. One representative experiment out of three is shown. **B**. Quantification of the intensity of the Ub conjugates (dashed rectangle) and free Ub (dashed square) identified in Figure 1A measured by densitometry of the respective areas. The fold change was calculated relative to the intensity at time 0. Mean ± SE of three experiments. **C**. Western blot illustrating the decrease of NEDD8 conjugates. The prominent band of approximately 100 kD corresponds to neddylated cullins. **D**. Quantification of the intensity of the NEDD8 conjugates and free NEDD8. Mean ± SE of three experiments.

**Figure 2:**
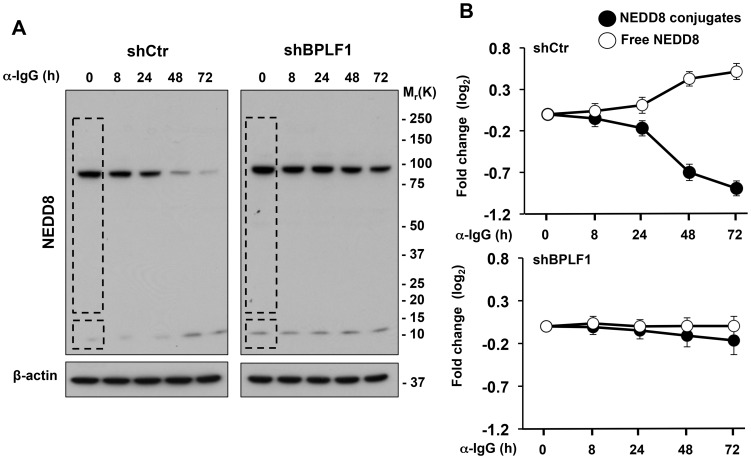
Protein deneddylation is dependent on the expression of BPLF1. The productive virus cycle was induced in Akata-Bx1 expressing a control or BPLF1-specific shRNA. Expression of the BPLF1 specific shRNA abolished the decrease of NEDD8 conjugates and accumulation of free NEDD8. **A**. Western blots of total cell lysates probed with a NEDD8 specific antibody. β-actin is shown as loading control. One representative experiment out of three is shown. **B**. Quantification of conjugated and free NEDD8. Mean ± SE of three experiments.

The Akata-Bx1 cell line was used to study the contribution of the Ub- and NEDD8-specific deconjugase activities of the EBV large tegument protein BPLF1 to the productive virus cycle. Treatment of Akata-Bx1 with anti-IgG antibodies promotes upregulation of the lytic cycle transactivator BZLF1, followed in temporal succession by the expression of immediate early, early and late viral gene products (Supplementary, Figure S1). Conjugated and free Ub and NEDD8 were quantified in western blots probed with specific antibodies using cells lysates made in the presence of cysteine protease inhibitors (Figure 1). The levels of conjugated and free Ub remained virtually unchanged over time (Figure 1A and 1B), whereas, consistent with the occurrence of deneddylation, the conjugated NEDD8 progressively decreased in parallel with increase of free NEDD8 (Figure 1C and 1D). In order to assess the involvement of BPLF1, a previously characterized specific shRNA [28] was transfected in Akata-Bx1 before induction of the productive cycle. As shown in Figures 2A and 2B, the effect was abrogated in cells expressing the BPLF1 specific shRNA, confirming that the phenotype is dependent on BPLF1 expression and supporting the conclusion that endogenous enzyme acts as a deneddylase during virus replication.

### BPLF1 selectively affects the expression and activity of nuclear CRLs

**Figure 3:**
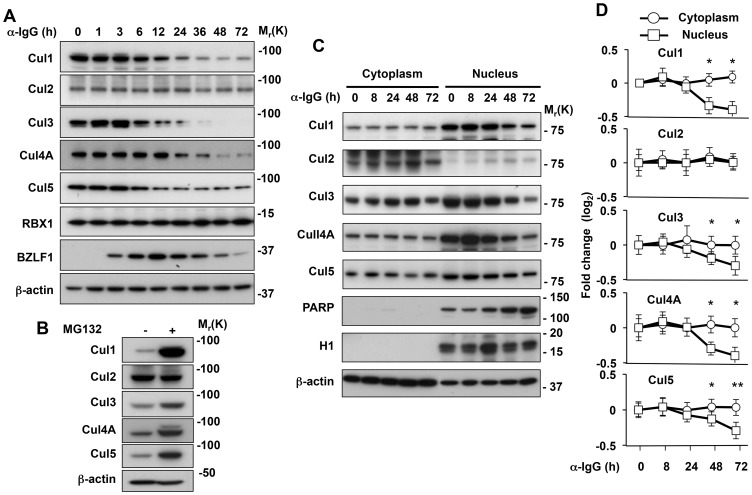
BPLF1 promotes the selective degradation of nuclear cullins by the proteasome. **A**. Cullins are selectively degraded during EBV replication. Western blots of cell lysates from induced Akata-Bx1 were probed with the indicated antibodies. One representative experiment out of three is shown. **B**. The cullins are degraded by the proteasome. Ten µM of the proteasome inhibition MG132 were added to one aliquot of Akata-Bx1 cells 48 h after induction and the cells were culture overnight before western blot analysis with the indicated antibodies. One representative experiment out of three is shown. **C**. Representative western blots illustrating the changes in expression levels of cytoplasmic and nuclear cullins. Subcellular fractionation was performed at the indicated time after induction and the efficiency of fractionation was confirmed by probing western blots with antibodies to PARP, histone H1 and β-actin. One representative experiment out of three is shown. **D**. Quantification of the fold change relative to the levels of expression at time 0. The mean ± SE of three experiments are shown. Significant differences between fold changes in the cytoplasmic and nuclear fractions are indicated * = p<0.01, ** = p<0.001.

Transfection of the catalytically active BPLF1 in HeLa cells promotes cullin deneddylation and their proteasomal degradation [29]. We asked therefore whether this phenotype is reproduced when the endogenous enzyme is expressed during virus replication. As illustrated by the representative western blot shown in Figure 3A, induction of the productive virus cycle was accompanied by a gradual decrease of the Cul1, Cul3, Cul4A and Cul5 specific bands in Akata-Bx1. This was not due to a global impairment of protein synthesis since neither Cul2, nor the CRL subunit RBX1 were affected. Furthermore, the decrease was rescued by treatment with MG132 confirming that cullins are degraded by the proteasome (Figure 3B). This finding is consistent with a scenario where the inactivation of CRLs by BPLF1-mediated deneddylation of cullins, and their subsequent proteasomal degradation, are key requirements for efficient virus replication. However, the failure to degrade Cul2 is surprising since the BPLF1 binding site on cullins is highly conserved [29]. To explore the possible cause of this observation, the abundance of Cul1, Cul2, Cul3, Cul4A and Cul5 was monitored over time in the nucleus and cytoplasm of the induced cells. The fractionation procedure was validated by probing of western blots with antibodies to PARP, histone H1 and β-actin. In line with their known subcellular localization, PARP and H1 were exclusively detected in the nuclear fractions, whereas β-actin was enriched in the cytoplasmic fraction (Figure 3C). Variable amounts of nuclear and cytoplasmic cullins were detected in untreated Akata-Bx1, with prevalent nuclear localization of Cul1, Cul3, Cul4A and Cul5, whereas Cul2 was detected almost exclusively in the cytoplasmic fraction (Figure 3C). Induction of the productive virus cycle was accompanied by a progressive decrease of nuclear pool of Cul1, Cul3, Cul4A and Cul5, whereas the amount of proteins detected in the cytoplasm remained unchanged throughout the observation period (Figure 3C and 3D). There was no detectable change in the expression of cytoplasmic Cul2, further supporting the conclusion that only nuclear cullins are affected.

**Figure 4:**
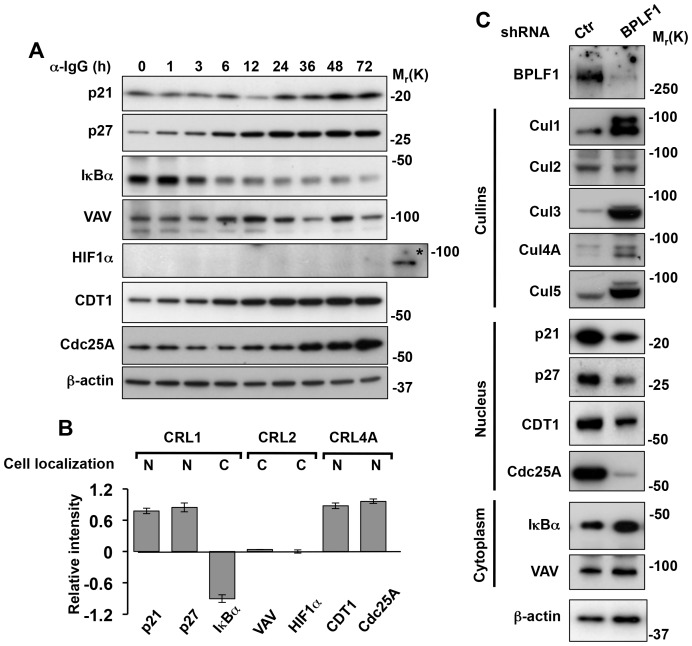
BPLF1 promotes the selective stabilization of nuclear CRL substrates. The expression levels of nuclear and cytoplasmic CRL substrates were monitored over time in induced Akata-Bx1 cells. **A**. Representative western blots illustrating the expression levels of CRL1 substrates: p21, p27 and IκBα, CRL2 substrates: VAV and HIF1α, and CRL4A substrates: CDT1 and Cdc25A. The expression level of β-actin is shown as loading control. As control, CRL2 was inactivated by exposing Akata-Bx1 to hypoxia, which resulted in stabilization of HIF1α (indicated by an asterisk). One representative experiment out of three is shown. **B**. Changes in the expression of nuclear and cytoplasmic CRL substrates in induced Akata-Bx1. The intensity of the specific bands detected at time 0 and after induction for 48 h was quantified. The mean ± SE of the fold change in three independent experiments is shown. **C**. The selective degradation of nuclear cullins and stabilization of nuclear CRL substrates is dependent on BPLF1. The productive cycle was induced in Akata-Bx1 transfected with a control or BPLF1 specific shRNA and cell lysates collected after 48 h were probed as indicated. One representative experiment out of three where each cullin and CRL substrate was tested is shown.

We then monitored the abundance of known nuclear and cytoplasmic CRL substrates. In agreement with the destabilization of the ligases, induction of the productive cycle was accompanied by the accumulation of several nuclear substrates of CRL1 and CRL4A, including p21, p27, CDT1 and Cdc25A, whereas two cytosolic substrates of CRL2, the Rho GTP exchange factor VAV [30], and the hypoxia induced factor HIF1α that is continuously degraded in normoxic conditions [31], were not affected (Figure 4A and 4B). IκBα, a cytosolic substrate of CRL1-βTRCP, was progressively degraded, confirming that the ligase is inactivated only in the nucleus. The involvement of BPLF1 in the degradation of nuclear cullins and stabilization of nuclear CRL substrates was confirmed by shRNA knockdown (Figure 4C). Thus, expression of the BPLF1 specific shRNA rescued the downregulation of Cul1, Cul3, Cul4A and Cul5 and promoted destabilization of their nuclear substrates p21, p27, CDT1 and Cdc25A, whereas the levels of Cul2, VAV and HIF1α (not shown) remained unchanged. Interestingly, the levels of IκBα were significantly increased in cells expressing the BPLF1 specific shRNA suggesting that the viral protein may indirectly regulate the activity of NFκB.

### CAND1 rescues the degradation of cullins and inhibits virus replication

**Figure 5:**
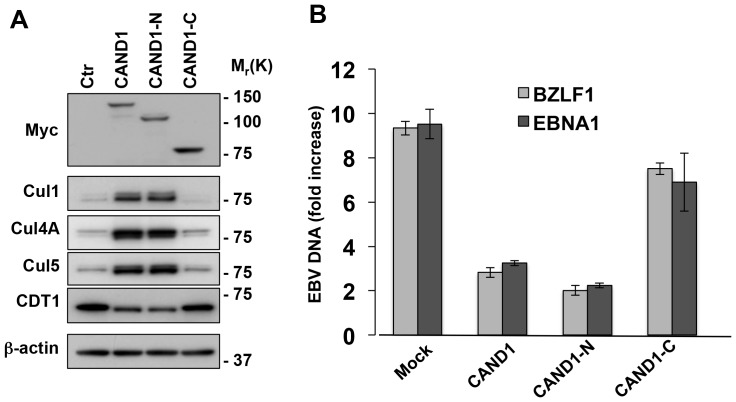
CAND1 rescues the degradation of cullins and inhibits virus replication. **A**. The degradation of cullins is rescued in cells transfected with the full-length and N-terminus of CAND1. The productive cycle was induced in Akata-Bx1 transfected with plasmids expressing the full-length, N-terminal or C-terminal domain of CAND1 or, as control, the empty vector. Twenty-four after transfection the cells were analyzed in western blots with the indicated antibodies. One representative experiment out of three is shown. **B**. CAND1 and the CAND1 N-terminus inhibit virus replication. The amount of EBV DNA was quantified by Q-PCR specific for unique sequence in the BZLF1 and EBNA1 genes. The mean ± SE of three independent experiments is shown.

We have previously shown that the capacity of BPLF1 to promote the deneddylation and degradation of cullins is dependent on binding to cullins at a site overlapping with the binding site of the regulator CAND1. The interaction is inhibited by overexpression of the N-terminus of CAND1, which rescues cullin deneddylation and degradation [29]. In order to assess whether this regulatory interaction may operate during virus replication, the productive cycle was induced in Akata-Bx1 cells transiently transfected with plasmids expressing Myc-tagged CAND1 or the CAND1 N-terminus that compete for BPLF1 binding to cullins, or, as controls, the CAND1 C-terminus that binds to the opposite end of the cullin scaffold, and the empty vector (Figure 5A). Expression of comparable amounts of the transfected proteins was confirmed in western blots probed with a Myc-specific antibody (Figure 5A, upper panels). In line with the above documented effects, low levels of Cul1, Cu4A and Cul5 and high levels of the CRL substrate CDT1 were detected upon induction of the productive cycle in cells transfected with the empty vector. The degradation of cullins and stabilization of CDT1 were reversed in cells expressing the full length or the N-terminus of CAND1 that compete for binding of BPLF1, whereas the C-terminus of CAND1 had no effect (Figure 5A). To assess the functional significance of this finding, the efficiency of viral DNA replication was measured by Q-PCRs in Akata-Bx1 transfected with CAND1 or the deletion mutants using primers specific for unique sequences in the BZLF1 and EBNA1 coding genes (Figure 5B). Induction of the productive cycle was associated with more than 10-fold increase of viral DNA content in cell transfected with the empty vector or the CAND1 C-terminus while viral DNA replication was strongly impaired in cells expressing the full-length CAND1 or the CAND1 N-terminus. It is noteworthy that only the full-length CAND1 that blocks both the N-terminal and C-terminal domains of cullins regulates the neddylation cycle in non-infected cells. Thus, the capacity of the N-terminal domain to fully reverse the inactivation of CRLs in infected cells is consistent with a mechanism of action based on inhibition of the binding of BPLF1 to cullins, and identifies CAND1 as a potent and specific cellular inhibitor of EBV replication.

### Cleavage by caspase-1 releases the catalytic N-terminus of BPLF1 and promotes its nuclear localization

**Figure 6:**
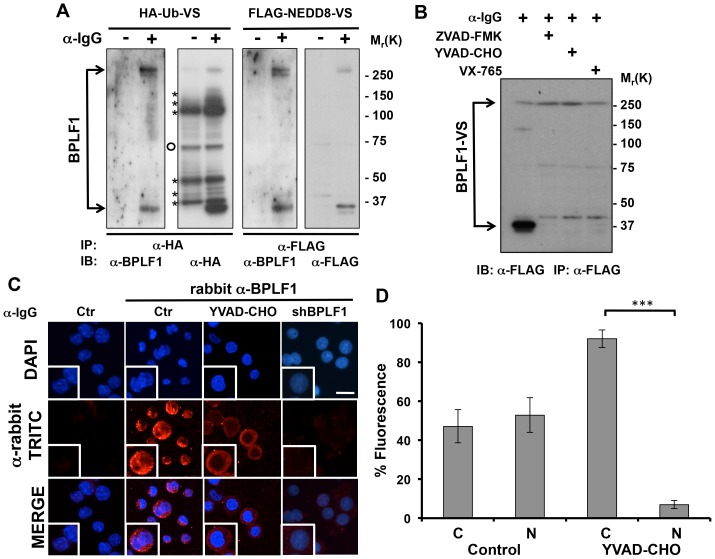
Cleavage by caspase-1 releases the catalytic N-terminus of BPLF1 and promotes its accumulation in the nucleus. **A.** A catalytically active N-terminal fragment of BPLF1 is produced during EBV replication. Lysates of control and induced Akata-Bx1 were labeled with the HA-Ub-VS and FLAG-NEDD8-VS functional probes and immunoprecipitated with HA- or FLAG-specific antibodies. Western blots were probed with antibodies to HA or FLAG and with an affinity purified rabbit serum raised against the N-terminus of BPLF1. Cellular DUBs labeled by the HA-Ub-VS functional probe are indicated by asterisks. A non-specific band of approximately 65 kD detected by the anti-HA antibody in both untreated and induced Akata-Bx1 is indicated by an empty circle. One representative experiment out of four performed with different batches of affinity-purified antibodies is shown. **B.** Inhibition of caspase-1 prevents the production of the catalytic N-terminal fragment. Lysates of Akata-Bx1 induced in the presence of the indicated caspase inhibitors were labeled with the FLAG-NEDD8-VS functional probe and FLAG immunoprecipitates were probed with a FLAG-specific antibody. One representative experiment out of three is shown. **C.** Inhibition of caspase-1 prevents the accumulation of BPLF1 in the nucleus. Fluorescence micrographs of Akata-Bx1 induced in the presence of absence of caspase-1 inhibitors stained with the affinity purified antibody to BPLF1. As controls induction was performed in Akata-Bx1 expressing a BPLF1 specific shRNA. Scale bar 2 µm. Representative cells are enlarged. **D.** Quantification of nuclear and cytoplasmic fluorescence in 50 cells from 2 independent experiments. The images were analyzed using the ImageJ software.

The large tegument proteins of herpesviruses are huge proteins predominantly localized in the cytoplasm of the infected cells where they play a key role in the delivery of viral DNA to the nuclear pore during primary infection and in the secondary envelopment and egress of mature virions [32]. However, the preferential effect on nuclear cullins and their substrates implies that the enzymatic activity of endogenous BPLF1 is compartmentalized to the nucleus. To find a possible cause of this puzzling observation, the subcellular localization of BPLF1 was investigated using a polyclonal rabbit serum raised against the catalytic N-terminus (amino acids 1-325). To confirm recognition of the active enzyme, lysates of untreated and induced Akata-Bx1 were labeled with the HA-Ub-VS and FLAG-NEDD8-VS functional probes that covalently bind to the catalytic cysteine. Western blots of anti-HA and anti-FLAG immunoprecipitates were then probed with antibodies to HA, FLAG and BPLF1. As illustrated by the representative blots shown in Figure 6A, the anti-HA and anti-FLAG antibodies detected two *de-novo* expressed enzymatic activities associated with polypeptides of >300 kD and approximately 38 kD in the lysates of induced cells. Probing of parallel blots with affinity purified antibodies to BPLF1 confirmed that the high molecular weight species corresponds to the full-length BPLF1, while the 38 kD species is likely to represent an approximately 25 kD N-terminal catalytic domain cross-linked to the 11 kD probe. Prediction algorithms were then used to screen the amino acid sequence of BPLF1 for the presence of cleavage sites for known cytosolic endo-peptidases. Several putative caspase-cleavage sites were identified in the first 2000 amino acids of BPLF1 (Supplementary, Figure S2). In particular, a high-score caspase-1 cleavage site in position Asp222 may generate a catalytically active fragment of the observed size. To test whether BPLF1 is cleaved by capsase-1, the productive cycle was induced in Akata-Bx1 treated with the pan-caspase inhibitor ZVAD-FMK, and the specific caspase-1 inhibitors YVAD-CHO and small molecule VX-765. Cell lysates collected after 48 h were labeled with HA-Ub-VS (not shown) and FLAG-NEDD8-VS. As shown in Figure 6B, both the full length and the 38 kD species were readily detected by the anti-FLAG antibody, although the 38 kD species was significantly stronger, which may be due to more efficient cross-linking of the probe or to poorer transfer of the high molecular weight full length enzyme. The intensity of the 38 kD fragment was strongly decreased when the induction was carried out in the presence of caspase inhibitors while the intensity of the full length species was slightly but consistently increased, confirming that the catalytic N-terminus of BPLF1 is cleaved by caspase-1.

Having established the specificity of the antibody, we then turned to investigate the subcellular localization of BPLF1. Staining of Akata-Bx1 with the affinity purified rabbit serum revealed a diffuse cytoplasmic and nuclear fluorescence 48 h after induction (Figure 6C), with essentially no background in cells stained with TRITC-conjugated secondary antibody alone. The specificity of the staining was confirmed by its virtual abrogation in induced cells expressing a BPLF1-specific shRNA. The nuclear fluorescence was virtually abolished when the induction was performed in the presence of the pan-caspase inhibitor (not shown) or the caspase-1 inhibitors YVAD-CHO (Figure 6C, 6D) and VX-765 (not shown). Thus, accumulation of the catalytic N-terminus of BPLF1 in the nucleus is dependent on cleavage of the cytosolic protein by caspase-1.

### Inhibition of caspase-1 prevents the inactivation of nuclear cullins and impairs viral DNA replication

**Figure 7:**
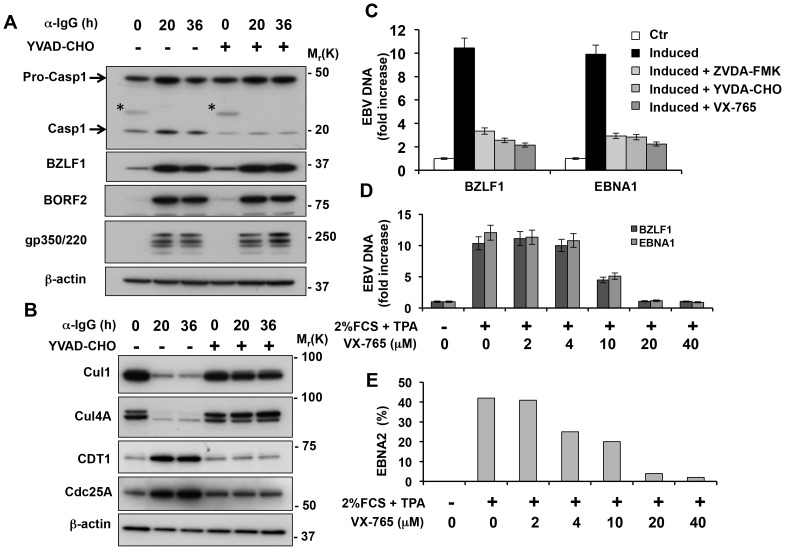
Inhibition of caspase-1 prevents the inactivation of nuclear CRLs and hampers EBV DNA replication. **A.** Caspase-1 is constitutively active in Akata-Bx1 and is further activated during virus replication but is not required for expression of the lytic cycle genes. Akata-Bx1 cells were induced in the presence or absence of caspase-1 inhibitors and western blots were probed as indicated. The asterisks in the caspase-1 blot indicate residual IgG heavy chains detected by the secondary antibody. One representative experiment out of three is shown. **B.** Inhibition of caspase-1 prevents the degradation of cullins and stabilization of CRL substrates. Western blots of cell lysates produced as described in Figure 7A were probed with the indicated antibodies. One representative experiment out of three is shown. **C.** Inhibition of caspase-1 hampers EBV DNA replication. The amount of viral DNA was measure by BZLF1 and EBNA1 specific Q-PCR in control induced Akata-Bx1 and cells induced in the presence of the indicated caspase-1 inhibitors. Mean ± SE of three experiments. **D.** The productive virus cycle was induced in the EBV positive B95.8 cell line by culture in medium supplemented with 2% FCS and 20 ng/ml TPA in the absence or presence of increasing concentrations of the caspase-1 inhibitor VX-765. The synthesis of viral DNA was monitored after 48 h by BZLF1 and EBNA1 specific Q-PCR. Mean ± SE of three experiments. **E.** Spent supernatants were collected after 2 weeks and the presence of infectious virus was measured by the induction of EBNA2 specific immunofluorescence in infected EBV negative BJAB cells. The mean % EBNA2 positive cells recorded in two independent experiments is shown.

Since the enzymatic activity of BPLF1 is required for efficient EBV DNA replication [27], [28], we tested whether the latter is affected by inhibition of caspase-1. In line with the constitutive IL-1 production of B-lymphoma cell lines [33], [34], two bands of approximately 48 kD and 20 kD, corresponding to the pro-caspase and active caspase-1, were detected in western blots of untreated Akata-Bx1 probed with a caspase-1 specific antibody (Figure 7A). The intensity of the 20 kD band increased upon induction of the productive cycle, which was prevented by addition of the caspase-1 inhibitors YVAD-CHO (Figure 7A) and VX-765 (not shown). Inhibition of caspase-1 did not affect the expression of the viral transactivator BZLF1, nor the subsequent expression of the early antigen BORF2 and late antigen gp350/220 (Figure 7A). Nevertheless, the treatment abrogated the BPLF1-dependent degradation of Cul1 and Cul4A and consequent stabilization of the CRL substrates CDT1 and Cdc25A (Figure 7B). In line with the requirement of CDT1 stabilization for efficient viral DNA replication [28], the yield of viral DNA was significantly impaired (Figure 7C). Similar levels of inhibition were achieved in cells treated with the pan-caspase inhibitor ZVAD-FMK and the caspase-1 specific inhibitors YVAD-CHO and VX-765. Thus, caspase-1 appears to be the sole responsible for BPLF1 processing.

In the final set of experiments we asked whether the effect of caspase-1 is restricted to Akata-Bx1 cell line. To this end, the productive virus cycle was induced by culturing the prototype EBV producer cell line B95.8 in medium supplemented with 2% FCS, 20 ng/ml TPA in the presence or absence of increasing concentrations of VX-765. As shown in Figure S4, the marmoset cell line expresses a conserved caspase-1 species that is detected by the cross-reactive antibody used in our experiments, and the intensity of a polypeptide of approximately 20 kD, corresponding to the active caspase-1, increased upon induction of the productive cycle. Inhibition of caspase-1 activity by addition of VX-765 resulted in a dose-dependent inhibition of viral DNA synthesis, with maximal inhibition observed in the presence of 20 µM VX-765 (Figure 7D). This was paralleled by a corresponding dose-dependent inhibition of the release of infectious virus assessed by the capacity of spent supernatants to induce the expression of EBNA2 in the EBV negative BJAB cell line (Figure 7E).

## Discussion

Our present study addresses an ongoing debate on the contribution of the deneddylase encoded in the large tegument protein of herpesviruses to virus replication, and provides a clear example of how, under physiologic conditions of expression, compartmentalization and binding to relevant substrates may determine the function of an enzyme with double specificity for both Ub and UbL conjugates.

Several lines of evidence support the notion that endogenously expressed BPLF1 promotes EBV replication by acting as a deneddylase. The recombinant enzyme has potent ubiquitin deconjugase activity *in vitro*, and overexpression of the catalytic N-terminus induces a global decrease of ubiquitin conjugates in transfected cells [24], [28]. However, induction of the productive cycle was not accompanied by significant changes in the amount of ubiquitinated proteins or free ubiquitin in Akata-Bx1, whereas neddylated proteins decreased and free NEDD8 increased in a BPLF1-dependent manner (Figures 1 and 2). While inconsistent with the potent deconjugase activity of BPLF1 in experimental settings, our failure to detect appreciable change in the global levels of ubiquitination during productive infection does not formally exclude that the viral enzyme might selectively target few ubiquitinated substrates. Yet, it should be stressed that evidence for the capacity of BPLF1 to deubiquitinate specific substrates, such as the EBV ribonucleotide reductase [35], PCNA [26] and TRAF6 [27], was in all cases obtained by overexpressing the catalytic N-terminus in transfected cells. In the experiments of Saito et al. reconstitution of the viral enzyme in cells infected with a mutant EBV strain that lacks BPLF1 was associated with deubiquitination of TRAF6 [27]. However, induction of the productive virus cycle was associated with strong TRAF6 deubiquitination also in the absence of BPLF1, suggesting that other factors are primarily responsible for this effect and for the subsequent downregulation of NF-κB target genes. This interpretation is also supported by our findings that the NFκB inhibitor IκBα was stabilized upon silencing of the endogenous BPLF1 in induced Akata-Bx1 (Figure 4C), which may be explained by failure to phosphorylate IκBα due to a BPLF1-independent inhibition of TRAF6 signaling associated with replicative cycle.

Similar to the effect of BPLF1 in transfected cells [28], [29], we found that endogenously expressed BPLF1 is required for the selective degradation of cullins in productively infected cells and for the stabilization of several CRL substrates that regulate the cell cycle and facilitate EBV DNA replication (Figures 3 and 4). The reversion of this phenotype by overexpression of the CRL regulator CAND1 (Figure 5) strengthens the notion that BPLF1 plays a key role in cullin deneddylation and degradation under physiological conditions of expression. We have previously reported that the conserved N-terminal domains of BPLF1 and CAND1 share a binding site on the C-terminus of cullins [29]. CAND1 regulates the activity of CRLs by sequestering cullins that are deneddylated after substrate ubiquitination, which promotes the exchange of substrate-adaptor modules, broadening the substrate repertoire and allowing rapid adaptation to a variety of metabolic conditions [36]. Cullins are the only known binding partners of CAND1 and it is therefore reasonable to assume that the reduced EBV DNA replication in cells overexpressing CAND1, and in particular the truncated CAND1 N-terminus that lacks the protein exchange function of the intact protein, is due to inhibition of the binding of BPLF1 of cullins. Collectively these findings support the notion that binding to the neddylated substrate determines the activity of this potentially promiscuous enzyme under physiological conditions of expression. This has two important implications. First, it underscores the possibility of experimental artifacts due to altered stoichiometry of the interacting partners in transfected cells. More importantly, it emphasizes the likelihood that interference with binding may have substantial downstream effects. In the case of EBV infection this could offer a new target for specific inhibition of virus replication.

We have shown that endogenously expressed BPLF1 acts on nuclear cullins and stabilizes nuclear CRL substrates while cytosolic CRLs and their substrates are not affected. This nuclear compartmentalization is dependent on cleavage of the catalytic N-terminus by caspase-1 (Figure 6). The processed fragment does not contain a putative nuclear localization signal but the size is sufficiently small for free diffusion through the nuclear pore that accommodates particles of up to 40 kD. Processing of BPLF1 is likely to be a key regulatory event in virus replication. It is noteworthy that a catalytically active N-terminal fragments of the BPLF1 homolog UL36 has been detected in cells infected with HSV1 [17], and preliminary results suggest that blockade of caspase-1 has a comparable inhibitory effect on HSV1 replication (Gastaldello et al. unpublished). Although processing of the tegument protein was not detected in cells infected with HCMV [19], [20], KSHV [37] or MHV-68 [38], and the full length enzymes encoded by these viruses are active DUBs, we have previously shown that the catalytic N-terminus of KSHV and MHV-68 share the cullin-binding capacity of BPLF1 and inactivate CRLs in transfected cells [28], [29]. It remains to be seen whether the failure to detect processing of some tegument proteins is explained by different experimental procedures or whether it might reflect true differences in the interaction of these viruses with the infected cells. In the case of BPLF1, it is tempting to speculate that, in addition to facilitating nuclear accumulation, cleavage of the catalytic N-terminus may also activate the viral enzyme. This possibility is supported by the observation that the band corresponding to the processed BPLF1 bound to the Ub-VS and NEDD8-VS functional probes was significantly stronger than the full length protein in lysates of induced cells (Figure 6A and 6B). Furthermore, cullins were not degraded in the cytosol, suggesting that the cytosolic enzyme is either inactive or cannot reach its targets. It is also possible that, while acting as a deneddylase in the nucleus, BPLF1, or perhaps the unprocessed form of the enzyme, may act as an ubiquitin-specific deconjugase in the cytoplasm of the infected cells. Experimental testing of this possibility remains an interesting focus for future work, pending the identification of substrates that are affected under physiologic conditions of expression. In this context it is noteworthy that cytosolic tegument proteins associated with the virion play important roles in the early and late phases of infection by contributing to the delivery of viral DNA to the nuclear pore and to the secondary envelopment and egress of mature virions [32]. Conceivably, a different set of cellular and viral substrates may be affected during these phases of the infection.

An unforeseen outcome of our study is the demonstration that the infected cell may regulate the efficiency of virus replication via caspase-1 mediated processing of BPLF1. Caspase-1 is well known for its role as the converging target of danger signals such as physical stress, extracellular ATP, bacterial and viral products, that are detected in the cytosol by sensing molecules and adaptors that promote the assembly of a multisubunit complex known as the inflammasome [39]. The inflammasome triggers the self-activation of caspase-1, which in turn mediates the maturation of pro-inflammatory cytokines like interleukin (IL)-1β and IL-18, and executes a rapid program of cell death known as pyroptosis [40]. Additional cellular substrates of caspase-1 include the Sterol Regulatory Element Binding Proteins (SREBPs) that are activated following changes in intracellular ions [41]. Thus, caspase-1 plays pleyotropic roles in the activation of innate and adaptive immune responses as well as in processes that link changes of the intracellular environment with lipid metabolism, membrane biogenesis and cell survival. Many viruses are known to inhibit the inflammasome or directly block the activity of caspase-1 in order to counteract antiviral responses [42]. Our findings highlight a previously unrecognized role of the cellular response to danger signals triggered by EBV reactivation in promoting rather than inhibiting virus replication. This further illustrates the complexity and multilayer regulation of the interaction of EBV with its host where the capacity of the virus to adapt to and exploit physiologic cellular responses underlies the establishment of life-long persistent infections.

## Materials and Methods

### Chemicals

DL-Dithiothreitol (DTT, D0632), *N*-Ethylmaleimide (NEM, E1271), Phenylmethanesulfonylfluoride (PMSF, P7626), Iodoacetamide (I1149), IGEPAL CA-630 (NP40, I3021), Sodium deoxycholate monohydrate (DOC, D5670), Triton X-100 (T9284), bovine serum albumin (BSA, A7906), 1,10-phenanthroline (o-phe, P9375), Sodium dodecyl sulfate (SDS, L3771), Tween-20 (P9416), Ethylenediaminetetraacetic acid disodium salt dehydrate (EDTA-E4884), Trizma base (Tris, 93349), Sodium butyrate (NaBut, B5887), Monoclonal Anti-HA conjugated agarose (A2095), Anti-FLAG conjugated agarose (A2220), Influenza hemagglutinin (HA) peptide (I2149), 3xFLAG peptide (F4799) were from Sigma Aldrich (St. Louis, MO). 12-O-Tertadecanoylphorbol-13-Acetate (TPA, 4174) was from Cell Signaling Technology (Danvers, MA). Complete protease inhibitors cocktail tablets (protease inhibitors) from Roche Diagnostic (Mannheim, Germany). HA-Ubiquitin-Vinyl Sulfone (HA-Ub-VS, U-212), FLAG-NEDD8-Vinyl Sulfone (FLAG-NEDD8-VS, UL-803) from Boston Biochem (Boston, MA). ZVAD-FMK, General Caspase Inhibitor (550377) was from BD Biosciences (Bedford, MA). Caspase-1 inhibitor I, cell-permeable (400011) was from EMD Millipore (Billerica, MA). The caspase-1 inhibitor VX-765 (CT-VX765) was from ChemieTek (Indianapolis, IN).

### Antibodies

Antibodies and their suppliers were: β-actin (AC-15, 1∶5000), RBX1 (1∶1000) from Sigma Aldrich; CDT1 (sc-28262, 1∶1000), Cdc25A (sc-7157, 1∶1000), EBV EA-R p85 (BORF2, sc-56979, 1∶1000), HIF1α (H206, 1∶1000) and EBV zebra (BZLF1, sc-53094), from Santa Cruz Biotechnology (Santa Cruz, CA); Cul1 (ab53049, 1∶1000), Cul2 (ab1870, 1∶1000), Cul3 (ab72187, 1∶1000), Cul4A (rabbit polyclonal ab2618, 1∶10000), Cul5 (ab33053, 1∶1000), Histone H1 (ab62884, 1∶500) and VAV-GEF (ab40875, 1∶1500) from AbCam (Cambridge, MA); hCAND1 (MCA4466Z, 1∶1000) from AbD Serotec (Oxford, UK); p21/Cip1/WAF1 (610233, 1∶1000) from BD Bioscience (Bedford, MA); Ubiquitin (Z0458, 1∶5000), p27/Kip1 (M7203, 1∶1000) and anti-human IgG polyclonal Rabbit antibodies (A0423, 1∶50) from DAKO (Glostrup, Denmark); NEDD8 (1∶1000) from Millennium Pharmaceuticals (Takeda Oncology Co, Cambridge, MA); Caspase-1 (06-503, 1∶1000) from EMD Millipore (Billerica, MA); Caspase-3 (06735, 1∶1000), Caspase-9 (05572, 1∶1000) from Upstate Biotechnology (Lake Placid, NY); Poly-(ADP-ribose)-polymerase (PARP) (BML-SA250, 1∶1000) from Enzo Life Science (Lörrach, Germany). A mouse monoclonal antibody to EBV-gp350/220 was a kind gift of Jaap M. Middeldorp, CCA-VUMC, Amsterdam, The Netherlands. A polyclonal rabbit antibody to BPLF1 was generated by immunization with purified GST-BPLF1 amino acid 1-325 (ASLA Biotech, Riga, Latvia). The antibody fraction was affinity purified from serum obtained from the third immunization, by NHS-activated sepharose (17-0906-02, GE Healthcare, Uppsala, Sweden) conjugated with HIS-BPLF1 1-325. After elution with 2M Glycine-HCl pH 2.2, and neutralization with 1 M Tris base, the antibody was used in western blot at dilution 1∶5000 and in immunofluorescence at dilution 1∶100.

### Plasmids and recombinant lentiviruses

Plasmids encoding a BPLF1 specific shRNA [28], and Myc-tagged full-length human CAND1, the CAND1 C-terminus (CAND1-C) and N-terminus (CAND1-N) [29] were described previously. Recombinant lentiviruses were produced in HEK293T cells transfected with the recombinant pLKO.1 expressing a control scrambled or BPLF1-specific shRNA and the packaging plasmids, psPAX2 and pMD2.G [43] (Addgene, Dr. Didier Trono, EPFL Lousanne, Switzerland) by Calcium Phosphate precipitation and supernatant containing viral particles was collected after 48 h. Virus titers were assessed by QuickTiter lentivirus titer kit, a HIV p24 specific ELISA (Cell Biolabs Inc., San Diego, CA).

### Cells line and transfection

Akata-Bx1 cells that carries a recombinant EBV where the thymidine kinase gene was replaced by a CMV immediate early promoter driven GFP [44] were cultured in Roswell Park Memorial Institute Medium (RPMI-1640, R8758 SIGMA-Aldrich, UK), supplemented with 10% Fetal Calf Serum (10270, GIBCO-Invitrogen, Carlsbad CA), Penicillin-Streptomycin (P0781, SIGMA-Aldrich, UK), L-Glutamine (G7513, SIGMA-Aldrich, UK) (complete medium) and Geneticin (10131-027, 200 µg/ml, GIBCO-Invitrogen, Carlsbad CA). Infection with recombinant lentiviruses was performed at m.o.i. 2.55E10 in the presence of 8 mg/ml Polybrene (AL-118, Sigma Aldrich). Induction of the productive virus cycle was initiated 24 h after infection. The cells were transfected with the Cell Line Nucleofector Kit V (AMAXA, Lonza Group, Ambroise, France).

### Induction of the EBV productive cycle, quantification of viral DNA and titration of infectious virus

The productive virus cycle was induced in Akata-Bx1 by incubating cell pellets for 1 h at 37°C with polyclonal rabbit anti-human IgG (1∶50, DAKO, Denmark) and monitored by the increased of GFP fluorescence or by probing western blots with antibodies specific for the EBV transactivator BZLF1. For induction of the productive cycle in the B95.8 cell line the cells were placed in a six well plate at the density of 5×10^4^ cells/ml in 5 ml medium supplemented with 2% FCS and 20 ng/ml TPA and the spent supernatant was harvested after 2 weeks [45]. Where indicated, the caspase-1 inhibitor VX-765 was added to the culture medium. Quantitative-PCR was performed on DNA extracted from the cell pellets and culture supernatant. Total DNA was purified with the DNAZol reagent (Invitrogen) and EBV DNA content was assayed by qPCR with the KAPA SYBR FAST qPCR Kit (KK4604, KAPA Biosystems, Cape Town, South Africa) and an AbiPrism 7000 Sequence detection system using 100 ng of DNA and the probes: EBNA1: 5′GGACGTGGAGAACAGTCATC3′/5′CACTCCTGCCCTTCCTCACC3′, product 364 bp; BZLF1: 5′CACTACCAGGTGCCTTTTGT3′/5′GAGACTGGGAACAGC TGAGG3′, product 364 bp; GAPDH: 5′AAGGTCGGAGTCAACGGATT3′/5′CTCCTGGAAGATGGTGATGG3′, product 224 bp. The cycling conditions were: initial step 50°C 2 min, denaturation 95°C 10 min, followed by 40 cycles of 95°C for 15 seconds, 60°C for 1 min, 95°C for 15 seconds, 60°C for 20 seconds and 95°C for 15 seconds. A final extension for 10 min at 72°C and melting curve between 65°C to 90°C, 1°C/second transition were incorporated. Optical raw data were exported to Microsoft Excel for analysis. All qPCR reactions were performed in triplicate and Ct values were averaged. The fold change in the target gene relative to the GAPDH endogenous housekeeping control gene is determined by: Fold Change = 2^−Δ (ΔCt)^ where ΔCt = Ct_target_ - Ct_GAPDH_ and Δ(ΔCT) = ΔCt_induced_ - ΔCt_control_, according to the Minimum Information for Publication of Quantitative Real-Time PCR Experiments (MIQE) guidelines. Production of infectious virus was induced by culturing the B95.8 cell line in medium supplemented with 2% FCS and TPA in the presence or absence of the indicated concentrations of the caspase-1 inhibitor VX-765. The presence of infectious virus in spent supernatants of the B95.8 cell line was assayed by infection of the EBV negative cell line BJAB. The percentage EBNA2 positive cells was assessed by immunofluorescence using the PE2 mouse monoclonal antibody (1∶150, NCL-EBV-PE2, Leica, Wetzlar, Germany) 48 h after infection.

### Immunofluorescence and cell cycle analysis

The subcellular localization of BPLF1 was investigated by immunofluorescence in Akata-Bx1 cells 48 h after induction in the presence or absence of caspase inhibitors. Eight ×10^4^ cells were deposited on glass slides by cytospin centrifugation, fixed with 4% paraformaldehyde and permeabilized with 0.5% v/v TritonX-100 in PBS for 20 min. The slides were then treated with blocking solution (0.5% BSA in PBS) for 2 h at RT and incubated with the anti-BPLF1 antibody O/N at 4°C followed by incubation for 1 h with TRITC conjugate anti-rabbit Ig (1∶200, R0270, DAKO). The slides were mounted with Vectashield mounting medium containing DAPI (H-1200, Vector Laboratories) and images were captured with a Zeiss LSM510 META confocal microscope and analyzed with the ImageJ 1.42q software (Wayne Rasband, National Institutes of Health, USA).

### Subcellular fractionation

Akata-Bx1 cells were washed in cold PBS containing 0.2 M freshly added iodoacetamide and resuspended in hypotonic lysis buffer (10 mM HEPES, pH 7.9, 1.5 mM MgCl_2_, 10 mM KCl, 0.1% NP-40, 1 mM DTT, 20 mM iodoacetamide, 1 mM ortho-phenantroline, 10 mM NEM and protease inhibitors cocktail. After incubation on ice for 30 min, the membranes were broken by passage through a 25–26 G needle and the nuclei were pelleted by centrifugation for 1 min at 10800 rpm. The supernatant was used as cytoplasmic fraction. The nuclei were washed three times with hypotonic buffer and lysed in buffer containing 50 mM Tris-HCl, pH 7.5, 150 mM NaCl, 1% NP40, 0.5% DOC and protease inhibitors cocktail. Protein concentration was measured with a Protein Assay kit (Bio-Rad Laboratories, CA).

### Immunoblotting and immunoprecipitation

Total cell lysates were prepared in lysis buffer (50 mM Tris-HCl pH 7.4, 150 mM NaCl, 1 mM DTT, 1 mM EDTA pH 8.0, 0.5% NP40, 0.01% SDS, 20 mM NEM, 1 mM ortho-phenantroline, protease inhibitors cocktail) and protein concentration was measured with a Protein Assay kit (Bio-Rad Laboratories, Solna, Sweden). Twenty µg of cell lysate were denatured for 10 min at 100°C in loading buffer and fractionated in acrylamide Bis-Tris 4–12% gradient gel (Invitrogen, Carlsbad, CA). After transfer to PVDF membranes (Millipore, Bedford, MA), the filters were blocked in PBS containing 0.1% Tween-20 and 5% non-fat milk or 3% BSA and incubated with the primary antibodies for either 1 h at room temperature or overnight at 4°C followed by incubation for 1 h with the appropriate horseradish peroxidase-conjugated secondary antibodies. The complexes were visualized by chemiluminescence (ECL, GE Healthcare, Uppsala, Sweden).

### Deconjugase activity assays

Deconjugase activity was assayed by labeling with the HA-Ub-VS and FLAG-NEDD8-VS functional probes (Boston Biochem, Boston, MA) as described [46]. Ten ×10^6^ cells were lysed in 300 µl of buffer containing 50 mM Tris-Cl pH 7.4, 50 mM NaCl, 1 mM DTT, 1 mM PMSF, 0.5% NP40, 250 mM Glucose, 5 mM MgCl_2_ (lysis and labeling buffer, LLB) followed by 20 strokes through a G30 needle. Functional labeling was performed by addition of 2.5 µg of HA-Ubiquitin-VS or FLAG-NEDD8-VS followed incubation for 45 min at 37°C. The cross-linked proteins were immunoprecipitated with Anti-HA-agarose or Anti-FLAG-agarose beads for 4 h at 4°C with rotation and eluted by competition with 25 µg/ml of the HA or FLAG peptides in LLB. Enzymatically active proteins were detected in western blots probed with anti-HA or anti-FLAG antibodies.

### Bioinformatics analysis

Putative caspase cleavage sites were searched in the BPLF1 amino acid sequence using the GraBCas software [47]. Sites with the highest probability of cleavage were identified by setting the cut-off scores to >15.

### Statistical analysis

Statistical analysis was performed using Student's t-test. P-values <0.05 were considered as significant.

## Supporting Information

Figure S1 **Kinetics of expression of Immediate Early, Early and Late antigens in induced Akata-Bx1.** Akata-Bx1 cells were treated for 1 h with anti IgG antibodies and western blots of cell collected at the indicated times were probed with antibodies to the Immediate Early antigen BZLF1, Early antigen BORF2 and Late antigen gp350/220. Western blots from one representative experiment are shown.
(TIF)

Figure S2 **Location of caspase cleavage sites predicted by GraBcas.** **A.** Domain Graph (DOG v2.0) diagram illustrating the location of the putative caspase cleavage sites in the first 2000 amino acids of BPLF1. The N-terminal domain including the catalytic Cys61 and His195 is marked in blue. The amino acid motifs of the predicted cleavage sites of caspase-1, -2, -6 and -8 are shown with the Aspartic acid colored in red. **B.** Cleavage position and GrabCas score of the caspase sites identified in the N-terminus of BPLF1.
(TIF)

Figure S3 **Effect of caspase-1 inhibition on the nuclear localization of BPLF1.** Representative localization profile of DAPI and TRITC fluorescence in induced Akata-Bx1 and cells treated with the caspase-1 inhibitor YVAD-CHO or BPLF1 specific shRNA. The BPLF1 specific fluorescence was homogeneously distributed in the nucleus and cytoplasm of untreated cells but was excluded from the nucleus of caspase-1 inhibitor treated cells. Background levels of BPLF1 fluorescence were observed in cells expressing a BPLF1 specific shRNA.
(TIF)

Figure S4 **Induction of the productive cycle promotes the activation of caspase-1 in B95.8 cells.** The productive cycle was induced in B95.8 cells by treatment with the indicated amounts of TPA or TPA and NaBut in medium containing 2% FCS. Induction of the EBV productive cycle was confirmed after one week by probing western blots of total cell lysates with antibodies specific for immediate early (BZLF1) early (BORF2) and late (gp350/220) antigens. Human caspase-1 specific antibodies detected a band of approximately 20 kD corresponding to the active caspase-1 in untreated cells and a stronger band was observed in the induced cells. The high levels of the active caspase-1 species detected in untreated cells is in line with the constitutive expression of the active enzyme in EBV transformed LCLs and may be partly explained by spontaneous entry into the productive cycle.
(TIF)
