# Supplementary figures and images for: Caspase-1 Promotes Epstein-Barr Virus Replication by Targeting the Large Tegument Protein Deneddylase to the Nucleus of Productively Infected Cells
Source: PLoS Pathog. 2013 Oct 10;9(10):e1003664. doi: 10.1371/journal.ppat.1003664 (PMC3795028; doi:10.1371/journal.ppat.1003664)

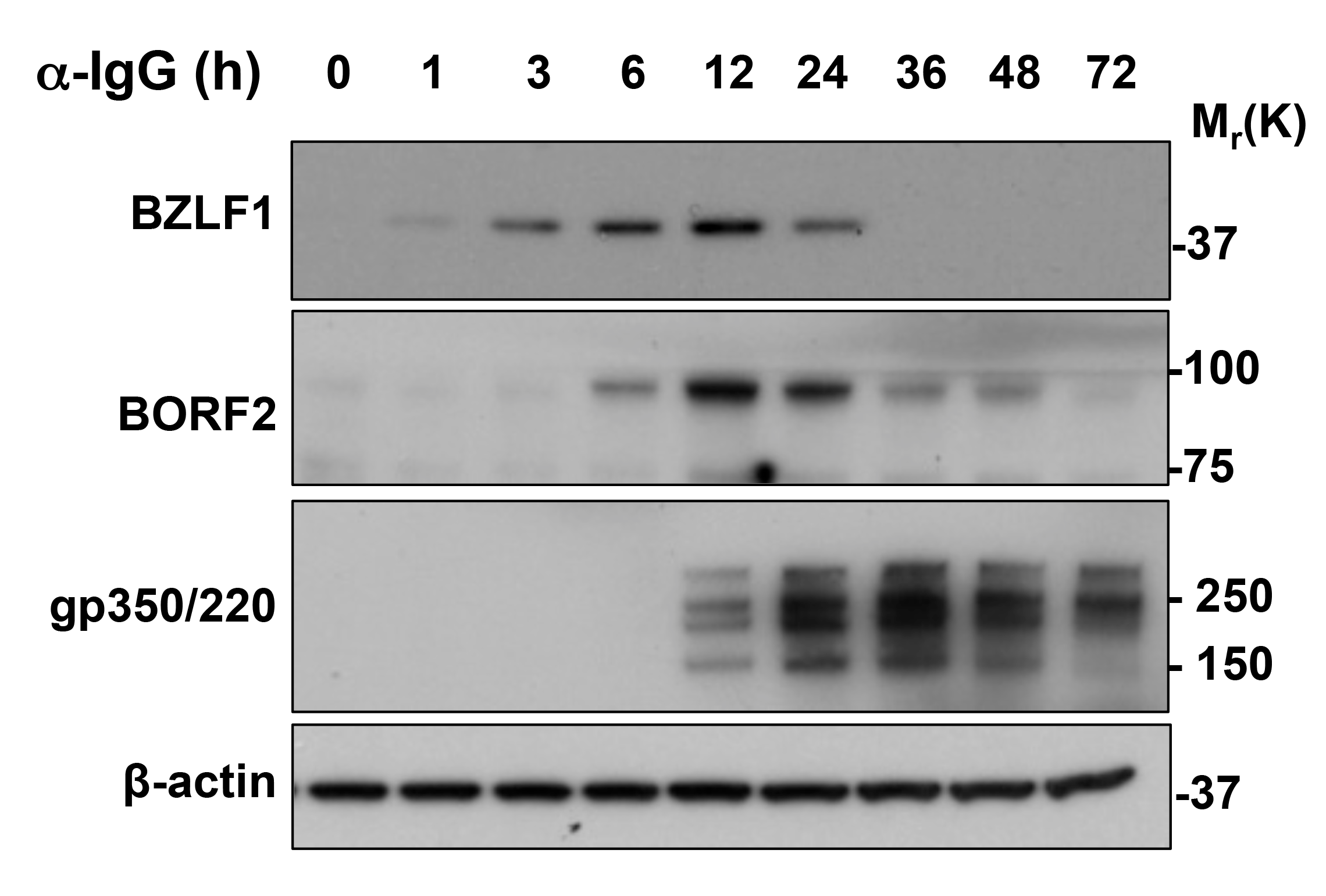

Supplement: Figure S1 — Kinetics of expression of Immediate Early, Early and Late antigens in induced Akata-Bx1. Akata-Bx1 cells were treated for 1 h with anti IgG antibodies and western blots of cell collected at the indicated times were probed with antibodies to the Immediate Early antigen BZLF1, Early antigen BORF2 and Late antigen gp350/220. Western blots from one representative experiment are shown. (TIF) [file ppat.1003664.s001.tif]

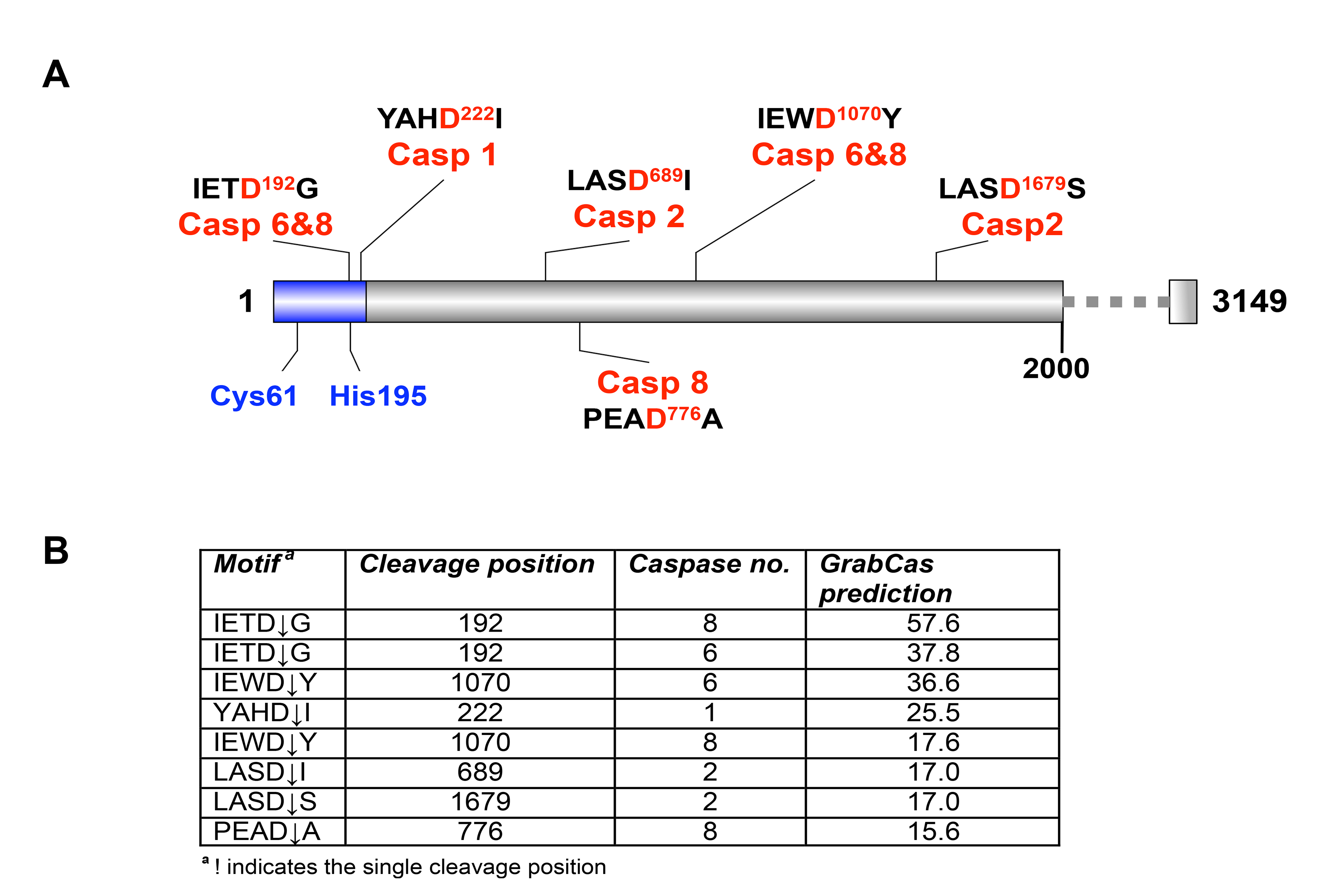

Supplement: Figure S2 — Location of caspase cleavage sites predicted by GraBcas. A. Domain Graph (DOG v2.0) diagram illustrating the location of the putative caspase cleavage sites in the first 2000 amino acids of BPLF1. The N-terminal domain including the catalytic Cys61 and His195 is marked in blue. The amino acid motifs of the predicted cleavage sites of caspase-1, -2, -6 and -8 are shown with the Aspartic acid colored in red. B. Cleavage position and GrabCas score of the caspase sites identified in the N-terminus of BPLF1. (TIF) [file ppat.1003664.s002.tif]

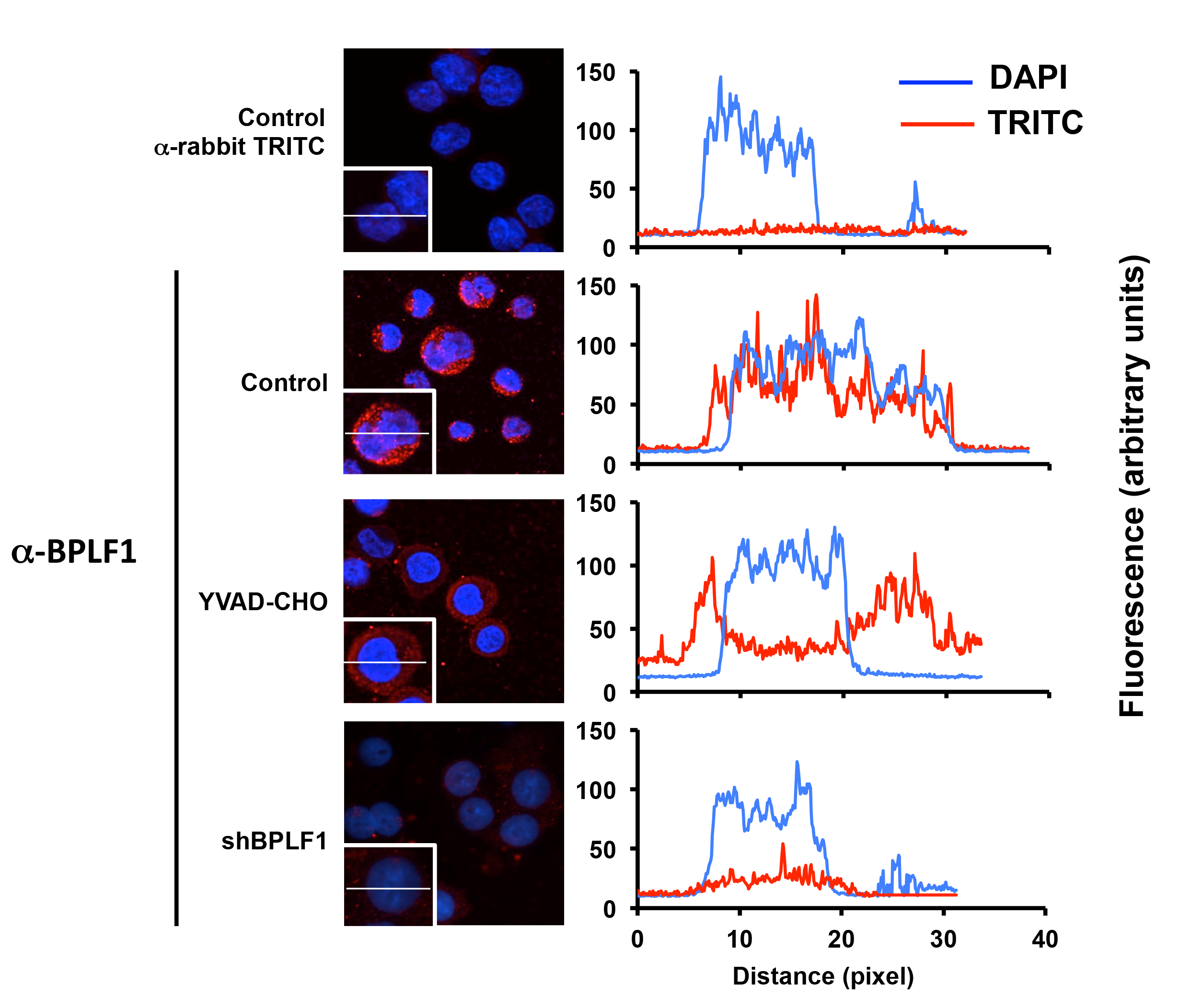

Supplement: Figure S3 — Effect of caspase-1 inhibition on the nuclear localization of BPLF1. Representative localization profile of DAPI and TRITC fluorescence in induced Akata-Bx1 and cells treated with the caspase-1 inhibitor YVAD-CHO or BPLF1 specific shRNA. The BPLF1 specific fluorescence was homogeneously distributed in the nucleus and cytoplasm of untreated cells but was excluded from the nucleus of caspase-1 inhibitor treated cells. Background levels of BPLF1 fluorescence were observed in cells expressing a BPLF1 specific shRNA. (TIF) [file ppat.1003664.s003.tif]

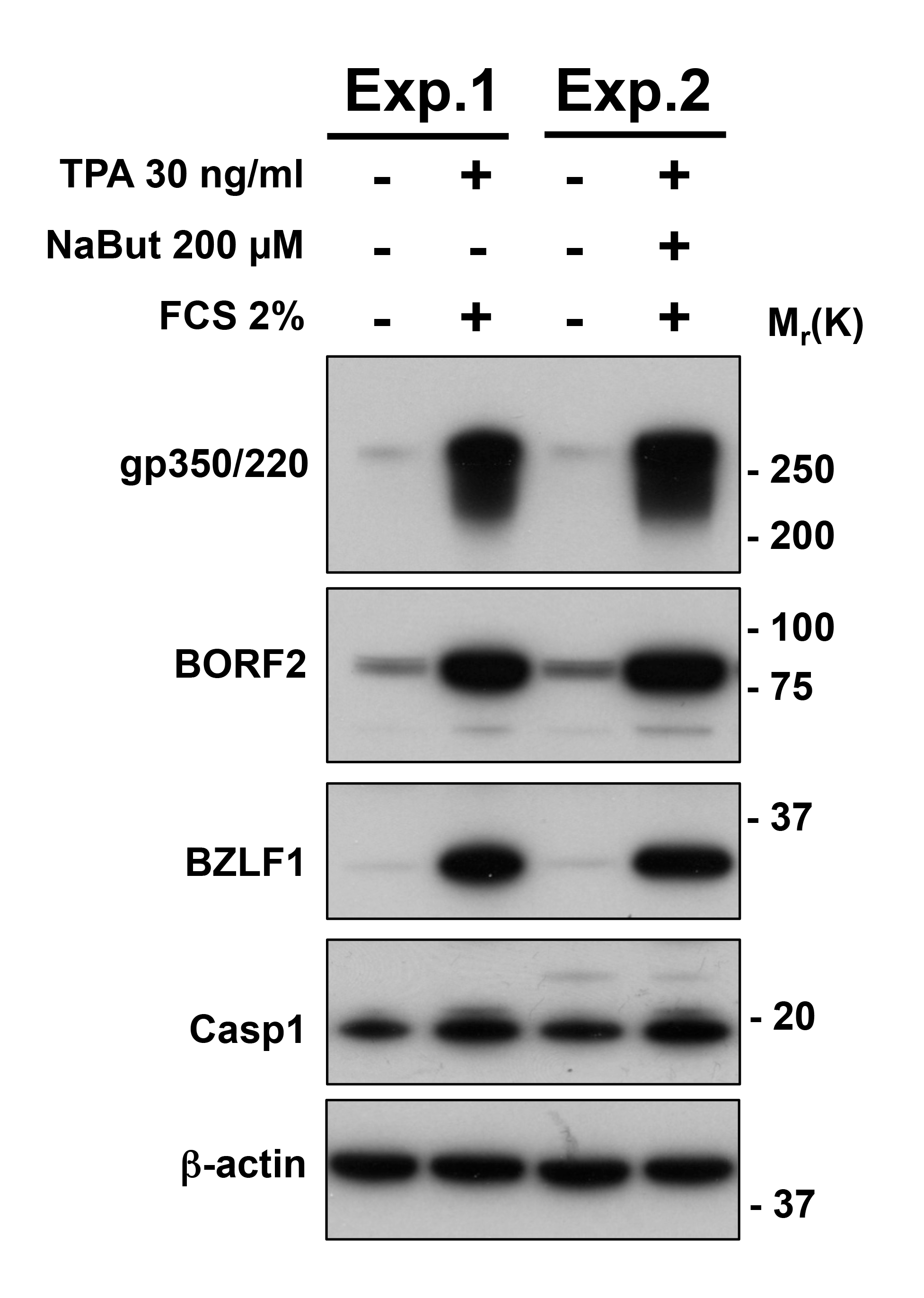

Supplement: Figure S4 — Induction of the productive cycle promotes the activation of caspase-1 in B95.8 cells. The productive cycle was induced in B95.8 cells by treatment with the indicated amounts of TPA or TPA and NaBut in medium containing 2% FCS. Induction of the EBV productive cycle was confirmed after one week by probing western blots of total cell lysates with antibodies specific for immediate early (BZLF1) early (BORF2) and late (gp350/220) antigens. Human caspase-1 specific antibodies detected a band of approximately 20 kD corresponding to the active caspase-1 in untreated cells and a stronger band was observed in the induced cells. The high levels of the active caspase-1 species detected in untreated cells is in line with the constitutive expression of the active enzyme in EBV transformed LCLs and may be partly explained by spontaneous entry into the productive cycle. (TIF) [file ppat.1003664.s004.tif]
